# Effects of Telemetric Interventions on Maternal and Fetal or Neonatal Outcomes in Gestational Diabetes: Systematic Meta-Review

**DOI:** 10.2196/24284

**Published:** 2021-08-27

**Authors:** Claudia Eberle, Stefanie Stichling

**Affiliations:** 1 Medicine with Specialization in Internal Medicine and General Medicine Fulda University of Applied Sciences Fulda Germany

**Keywords:** digital health, eHealth, gestational diabetes, systematic meta-review, telemedicine, telemetry, telemonitoring

## Abstract

**Background:**

In 2019, 1 of 6 births was affected by gestational diabetes mellitus (GDM) globally. GDM results in adverse maternal, fetal, and neonatal outcomes in the short and long term, such as pregnancy and birth complications, type 2 diabetes, metabolic syndrome, and cardiovascular disease. In the context of “transgenerational programming,” diabetes mellitus during pregnancy can contribute to “programming” errors and long-term consequences for the child. Therefore, early therapy strategies are required to improve the clinical management of GDM. The interest in digital therapy approaches, such as telemetry, has increased because they are promising, innovative, and sustainable.

**Objective:**

This study aimed to assess the current evidence regarding the clinical effectiveness of telemetric interventions in the management of GDM, addressing maternal glycemic control, scheduled and unscheduled visits, satisfaction, diabetes self-efficacy, compliance, maternal complications in pregnancy and childbirth, as well as fetal and neonatal outcomes.

**Methods:**

Medline via PubMed, Web of Science Core Collection, Embase, Cochrane Library, and CINAHL databases were systematically searched from January 2008 to April 2020. We included randomized controlled trials, systematic reviews, meta-analyses, and clinical trials in English and German. Study quality was assessed using “A MeaSurement Tool to Assess systematic Reviews” and “Effective Public Health Practice Project.”

**Results:**

Our search identified 1116 unique studies. Finally, we included 11 suitable studies (including a total of 563 patients and 2779 patient cases): 4 systematic reviews or meta-analyses (1 of high quality and 3 of moderate quality), 6 randomized controlled trials (2 of high quality and 4 of moderate quality), and 1 low-quality nonrandomized controlled trial. We classified 4 “asynchronous interventions” and 3 “asynchronous and real-time interventions.” Our findings indicate that telemetric therapy clearly improves glycemic control and effectively reduces glycated hemoglobin A_1c_ levels. Furthermore, in 1 study, telemetry proved to be a significant predictor for a better glycemic control (hazard ratio=1.71, 95% CI 1.11-2.65; *P*=.02), significantly fewer insulin titrations were required (*P*=.04), and glycemic control was achieved earlier. Telemetric therapy significantly reduced scheduled and unscheduled clinic visits effectively, and women were highly satisfied with the treatment (*P*<.05). From fetal and neonatal short-term outcomes, some improving tendencies in favor of telemetry were determined. No long-term outcomes were detected.

**Conclusions:**

Telemetric interventions clearly improved glycemic control, notably glycated hemoglobin A_1c_ levels, and reduced scheduled and unscheduled clinic visits effectively, which reinforces this digital approach in the treatment of GDM.

## Introduction

In 2019, 1 of 6 births was affected by gestational diabetes mellitus (GDM), according to the International Diabetes Federation [1]. Overall, some form of hyperglycemia was detected in approximately 16% of live births [1]. GDM is a major clinical health problem, with a lack of common global guidelines [2]. The reported cases of GDM have drastically increased worldwide [2]. According to the International Diabetes Federation in 2019, the prevalence of GDM ranged from 7.5% in Middle East and North Africa, 9.6% in Africa, 12.5% in Western Pacific countries, 13.5% in South and Central America, 16.3% in Europe, and 20.8% in North America and the Caribbean to 27.0% in South-East Asia, excluding countries with no estimates [3].

GDM is diagnosed in the second or third trimester of pregnancy and not overt diabetes prior to gestation [4]. The condition results in various adverse pregnancy outcomes [1]. For example, women with GDM have an increased risk of developing type 2 diabetes mellitus, metabolic syndrome, and coronary heart disease in the long term. Short-term consequences such as premature birth and pre-eclampsia can also occur [2,4,5]. According to the American Diabetes Association, there are also short-term consequences for the child, such as fetal anomalies, fetal demise, macrosomia, neonatal hypoglycemia, and hyperbilirubinemia as well as long-term consequences, such as an increased risk of obesity, hypertension, and type 2 diabetes in later life [4].

Influences during the pre- and perinatal period, among other factors, play a decisive role for health and illness in the course of later life [5]. Transgenerational programming (“fetal programming”), a perturbation during critical development phases (prenatal), can lead to a “programming error” in organ functions and metabolic regulation, on the basis of which diseases such as impaired glucose intolerance, non–insulin-dependent diabetes, hypertension, obesity, and cardiovascular disease, can develop in adulthood [5]. Diabetes during pregnancy can contribute to such programming errors and to long-term consequences for the child [5]. In this context, early therapy strategies are required to improve the clinical management of GDM effectively. GDM is limited in time; therefore, paltry time is available to detect and treat the condition [2]. As part of the clinical management of GDM, the American Diabetes Association recommends self-monitoring of fasting and postprandial blood glucose to accomplish metabolic control as well as lifestyle management, including physical activity, weight management, and medical nutrition treatment [2].

However, telemetric interventions provide new digital options to enhance clinical outcomes in GDM therapy. Interest in digital solutions such as telemetry is increasing because they are innovative and sustainable approaches. In telemetric interventions, patient data are collected remotely and transmitted via telecommunication systems to a health care provider [6]. Telematics, the science of telecommunication and informatics, was developed in the 1970s [7]. Over the years and with the advancement of technology, various digital concepts developed and expanded, such as telemedicine, eHealth, mHealth, and digital health [7]. Other reviews and meta-analyses reported positive outcomes of telemetry in GDM management [8,9]. However, evidence of the clinical effectiveness of telemetric interventions in GDM management is still lacking.

In this systematic meta-review, we aimed to assess evidence regarding the clinical effectiveness of telemetric interventions in the management of GDM to improve maternal, fetal, and neonatal short- and long-term outcomes to counteract transgenerational programming. We focused on the communication and interaction between health care professionals and patients and included different studies including randomized controlled trials (RCTs), systematic reviews (SRs), meta-analyses (MAs), and clinical trials.

## Methods

### Information Sources and Search Strategy

The systematic meta-review was based on the PRISMA (Preferred Reporting Items for Systematic Reviews and Meta-Analyses) guidelines [10]. We performed a comprehensive systematic search in different databases including Medline via PubMed, Web of Science Core Collection, Embase, Cochrane Library, and CINAHL. We selected the following keywords from the Medical Subject Headings and Embase subject headings databases and additionally searched them as title and abstract terms: “gestational diabetes,” “pregnancy diabetes mellitus,” “telemetry,” “telemonitoring,” and “telemedicine.” The search strategy in the databases is explained in Multimedia Appendix 1.

### Eligibility Criteria

The systematic literature search was limited to publications from January 2008 to April 2020, which target the clinical management of GDM. The telemetric interventions involved monitoring, including data transmission to health care providers and appropriate feedback to patients diagnosed with GDM (eg, web-based technologies, telephone calls, and video consultations). Furthermore, we included peer-reviewed studies in English and German and those with the following designs: RCTs, SRs, MAs, and clinical trials, including qualitative and quantitative studies.

We excluded studies that provided pooled data with other types of diabetes mellitus or with other digital applications and apps; those focused on the prevention, screening, or diagnosis of GDM; and those that described only the technologies. Furthermore, we excluded smartphone or mobile app–based interventions. Because of the different nature of these technologies, we examined them separately in another study.

### Study Selection

First, we conducted an extensive literature search that included patients with type 1 diabetes mellitus, type 2 diabetes mellitus, and GDM. After the elimination of duplicates, screening of titles and abstracts, appraisal of studies with full-text access for eligibility, and additional scrutiny of reference lists to identify further studies, we finally selected suitable studies that focused on GDM for this systematic meta-review. The study selection process is described in Multimedia Appendix 2.

### Data Extraction

The following data were extracted from the included studies: publication year, intervention duration, sample sizes, location, outcomes, key results, significant statistics, and conclusions.

### Data Synthesis and Analysis

For the synthesis and analysis of data from the included studies, we developed and applied a scheme that classified the interventions in accordance with the technologies they used. We generated 4 categories of interventions, based on the technologies used for communication between health care professionals and patients: (1) interventions with “real-time video communication,” including synchronous face-to-face communication using videoconferencing; (2) those with “real-time audio communication,” including synchronous contact through telephone calls; (3) those with “asynchronous communication,” including interaction via email, SMS text messaging, server or home gateway, and web-based platforms; and (4) those that combined “asynchronous and real-time communication.” In addition to this classification according to our scheme, we structured the studies by their designs and outcomes.

We also added up the number of participants, first including the number of unique patients with GDM in the clinical trials (excluding SRs and MAs) and then the number of patient cases based on outcomes wherein a patient has been accounted for multiple times (including SRs and MAs).

### Assessment of Risk of Bias

We assessed study quality by using 2 different tools because of variations in the design of the included studies. We used the valid and reliable instrument “A MeaSurement Tool to Assess systematic Reviews” [11] for evaluating SRs and MAs and “Effective Public Health Practice Project” [12] for appraising RCTs and non-RCTs. Both instruments classify the methodological quality ranging between “high” (or “strong”), “moderate,” and “weak” (or “low”).

## Results

### Study Selection and Characteristics

The extended literature search yielded 1647 studies, of which 1116 unique studies were screened on the basis of the defined eligibility criteria. After an additional search of reference lists, we identified 189 studies, of which 23 focused on type 1 diabetes mellitus, 99 on type 2 diabetes mellitus, 51 on mixed populations (type 1 and type 2 diabetes mellitus), and 11 on GDM. Finally, we selected 11 suitable studies on GDM in this systematic meta-review. Of them, 4 were SRs and MAs, 6 RCTs, and 1 was a non-RCT. Particularly among RCTs and non-RCTs (n=7), most of them were carried out in Europe (n=5, 51.1%) and 1 each in the United States, Canada, and Australia. Baseline characteristics of the studies are provided in Table 1, and a detailed description of the studies is provided in Multimedia Appendix 3.

Using the instruments, “A MeaSurement Tool to Assess systematic Reviews” and “Effective Public Health Practice Project,” we evaluated 3 studies (1 SR or MA and 2 RCTs) as being of high quality, 7 studies (3 SRs or MAs and 4 RCTs) of moderate quality, and 1 non-RCT of low quality. An overview of the quality assessments is provided in Multimedia Appendix 4.

Generally, the included studies involved 563 individual patients and 2779 patient cases. Owing to the high heterogeneity of the telemetric interventions, the SRs and MAs were not classified in accordance with their types of intervention. We identified 4 “asynchronous interventions” (web-based systems) and 3 “asynchronous and real-time interventions” (web-based systems and telephone communication). No studies were recognized for the previously defined categories “real-time audio interventions” and “real-time video interventions.”

**Table 1:** Baseline characteristics of the included studies (N=11).

Characteristics			Studies, n (%)
**All studies**			
	**Study design**		
		Systematic review or meta-analysis	4 (36.4)
		Randomized controlled trial	6 (54.6)
		Non-randomized controlled trial	1 (9.0)
	**Year**		
		2008-2011	2 (18.2)
		2012-2014	1 (9.0)
		2015-2017	5 (45.5)
		2018-2020	3 (27.3)
**Studies excluding systematic reviews and meta-analyses (n=7)**			
	**Location**		
		United States	1 (14.3)
		Canada	1 (14.3)
		Europe	4 (51.1)
		Australia	1 (14.3)
	**Intervention**		
		Asynchronous	4 (51.1)
		Asynchronous and real-time	3 (42.9)

### Synthesis of Results

A summary of the effects of maternal, fetal, and neonatal outcomes of each study is allocated in Multimedia Appendix 5.

#### Maternal Glycemic Control

*Glycated hemoglobin A_1c_ (HbA_1c_) values (4 studies)*. In total, the studies presented clear improvements in HbA_1c_ values through telemetric interventions. Ming et al [9] (moderate quality [MQ]) indicated an obvious significant reduction of –1.14% (mean difference [MD]) (95% CI –0.25 to 0.04; *P*=.01) in HbA_1c_ values among 196 patients in the intervention groups compared to the control groups (220 patients). Similarly, Rasekaba et al [8] (MQ) reported a clear enhancement (MD=–0.18%; 95% CI –0.50 to 0.14; *P*=.27) (144 patients). In contrast, Pérez-Ferre et al [13] (HQ) reported a minor deterioration in both groups (<5.8% among all women), and Given et al [14] (MQ) noted slightly lower HbA_1c_ values in the control group than in the intervention group. Both contrasting studies did not report *P* values and had comparatively smaller sample sizes (97 and 47 patients, respectively). In addition, Given et al [14] reported that slow and unreliable data transmissions (especially because of poor mobile reception in rural households that could not use a landline) affected the use of the telemetric system.

*Insulin dose (1 study)*. In general, Rasebaka et al [15] (MQ) reported that the use of telemetric approaches had positive effects on the insulin dose. The treatment group (61 patients) required significantly fewer median insulin titrations (4, IQR 13) than the control group (34 patients; 13, IQR 25; *P*=.04). Furthermore, optimal glycemic control was achieved among the intervention subjects (maximum dose of insulin) significantly quicker than among the control subjects (4.3 weeks vs 7.6 weeks; *P*<.001). Telemetry proved to be a significant predictor of better glycemic control (hazard ratio 1.71, 95% CI 1.11-2.65; *P*=.02).

*Gestational weeks at insulinitation (1 study)*. Pérez-Ferre et al [10] (HQ) reported explicitly earlier insulinitation in the intervention group (n=17) at 27.73 (SD 3.13) gestational weeks than in the control subjects (n=9) at 28.22 (SD 3.80) gestational weeks (*P*=.73).

#### Maternal Scheduled and Unscheduled Visits

*Face-to-face visits (6 studies)*. Almost all studies (83.3%) reported that the number of face-to-face clinic visits decreased explicitly. Pérez-Ferre et al [10,13] (HQ), Lemelin et al [16] (low quality [LQ]), and Caballero-Ruiz et al [17] (MQ) outlined large significant reductions ranging between 56.00% and 88.56% (*P*=.002 [10], *P*<.03 [13], *P*<.001 [16], and *P*<.01 [17]). Pérez-Ferre et al [13] reported an even greater reduction in insulin-treated patients (62% overall and 82% in insulin-treated patients; *P*<.03). Only 1 study [15] (MQ) reported the same number of visits in the intervention and control groups.

*Unscheduled visits (3 studies)*. Overall, most studies described substantially fewer unscheduled visits in the treatment groups. Rasekaba et al [8] (MQ) and Pérez-Ferre et al [10] (HQ) outlined significantly fewer unscheduled clinic visits (*P*=.03), with Rasekaba et al (SR and MA) [8] referring to the study by Pérez-Ferre et al [10]. Pérez-Ferre et al [10] reported even fewer visits in the subgroup of insulin-treated patients (intervention: 0.50, SD 0.73 vs control: 2.89, SD 1.05; *P*<.001).

*Obstetrical emergency visits (1 study)*. Lemelin et al [16] (LQ; 161 patients) revealed significantly (*P*=.014) and noticeably fewer patients with ≥1 visit to the obstetrical emergency clinic in the intervention group (2.0%, SD 2.3%) compared to the control group (3.0%, SD 3.0%).

#### Maternal Satisfaction and Diabetes Self-Efficacy

*Satisfaction (4 studies).* In general, all studies delineated that the intervention groups were highly satisfied. Fantinelli et al [18] (MQ) and Lemelin et al [16] (LQ) reported significantly higher satisfaction in the intervention groups (*P*<.001 [18] and *P*=.03 [16]). Lemelin et al [16] refers to the satisfaction with educational support. Given et al [14] (MQ) and Caballero-Ruiz et al [17] (MQ) consistently noted high satisfaction with the telemetric support (without significant statistics reported).

*Diabetes self-efficacy (2 studies)*. In total, both MQ reviews by Rasebaka et al [8] and Fantinelli et al [18] revealed higher scores in diabetes self-efficacy in the telemedical group than in the control group, but both referred to the same included study with significantly higher scores in 2 subscales (*P*=.039 vs *P*=.036).

*Compliance (3 studies).* Generally, the participants in the intervention groups were more compliant. Fantinelli et al [18] (MQ) examined several studies with a total of 401 patients and concluded that the intervention groups were more compliant (no significant statistics reported). According to Homko et al [19] (MQ), who examined an internet-based system with automated reminders, the integration of reminders significantly improved patients’ compliance in comparison with a previously conducted study without reminders (data sets transmitted: 17.4, SD 16.9 in the previous study to 35.6, SD 32.3 in this study; *P*<.01). In the study by Caballero-Ruiz et al [17] (MQ), slightly more blood glucose measurements were transferred in the treatment group (n=147.017) than in the control group (n=141.562) (*P*<.05).

#### Maternal Complications in Pregnancy

*Pregnancy-induced hypertension (4 studies)*. In general, 2 of 4 studies (MQ and LQ) reported an explicitly lower number of women with pregnancy-induced hypertension in groups receiving the telemetric intervention (1.3% in the intervention group vs 2.5% in the control group; *P*=.23 [16]; 0.0% in the intervention group vs 3.9% in the control group, significant statistics not reported [14]). In contrast, the HQ study by Pérez-Ferre et al [13] indicated more cases in the intervention group (4.1% int eh intervention group vs 0.0% in the control group; *P*=.50), but with a smaller sample size (n=97) than the other 2 trials (n=208). In addition, Raman et al [20] (HQ) calculated a risk ratio (RR) of 1.49 (95% CI 0.69-3.20) (n=275).

*Pre-eclampsia (4 studies).* One of the studies reported a clearly lower number of women diagnosed with pre-eclampsia in the intervention group (Given et al [14] [MQ]: 0.0% in the intervention group vs 3.9% in the control group; *P* value not available). In the other studies, the number of pre-eclampsia cases is either the same in both groups or in favor of the control group (Raman et al [20] [HQ]: RR 1.49, 95% CI 0.69-3.20 based on 4 RCTs; Lemelin et al [16] [LQ]: no cases in both groups; Homko et al [19] [MQ]: *P*=.07). In addition, Raman et al [20] noticed a very low quality of the examined 4 RCTs.

#### Maternal Complications in Childbirth

*Caesarean section rate (7 studies)*. Three (42.86%) MQ and LQ studies demonstrated positive tendencies in favor of the intervention group, but overall no significant effects were found [13,15-17,19,20]: Rasekaba et al 2015 [8] (MQ; 228 patients; odds ratio 0.48, 95% CI 0.10-2.35), Raman et al [20] (HQ; 5 RCTs with 478 patients; RR 1.05, 95% CI 0.72-1.53), Rasekaba et al [15] (MQ; *P*=.20), Pérez-Ferre et al [13] (HQ; *P*=.43), Lemelin et al [16] (LQ; *P*=.07), and Homko et al [19] (HQ; *P*=.30). Raman et al reported a very low quality of the 5 RCTs used to calculate the RR.

*Preterm delivery (<37 weeks; 4 studies)*. In 2 of 4 MQ studies, the number of preterm deliveries in the intervention group was distinctively lower (0% in the intervention group vs 8% in the control group; no *P* value reported [14]; 5.6% in the intervention group vs 13.2% in the control group; *P*=.30 [19]). With this outcome, it is striking that these 2 studies are assigned to the category “asynchronous and real-time communication,” whereas the 2 HQ and LQ studies with “asynchronous communication” had slightly more cases of premature birth in the intervention group (2.1% in the intervention group vs 2.0% in the control group; *P*=0.50; 3.8% in the intervention group vs 0.0% in the control group; *P*=0.08).

*Other complications (3 studies)*. With the outcomes “induction of labor” (Raman et al [20] [HQ], RR 1.06, 95% CI 0.63-1.77), “umbilical cord pathology” (Pérez-Ferre et al [13] [HQ], *P*=.50), “abruptio placentae” (Pérez-Ferre et al [13] [HQ], *P*=.50), and “chorioamnionitis” (Homko et al [19] [MQ], *P*>.99), the respective HQ and MQ studies were able to determine a very slightly positive trend in favor of the control group, with small sample sizes.

#### Fetal Short-term Outcomes

In the outcomes, “loss of fetal well-being” (Pérez-Ferre et al [13] [HQ], 6.1% in the intervention group vs 8.3% in the control group; *P*=.50) and “intrauterine death” (Given et al [14] [MQ], 0.0% in the intervention group vs 3.9% in the control group; significant statistics not reported), small positive tendencies in favor of the intervention groups were predominantly found.

#### Neonatal Short-term Outcomes

*Large for gestational age (LGA) (3 studies)*. In total, the study with “asynchronous communication” showed positive tendencies in favor of the intervention group, whereas the trial with “asynchronous and real-time communication” outlined a higher number of LGA cases in the intervention group (Pérez-Ferre et al [13] [HQ], 6.1% in the intervention group vs 8.3% in the control group; *P*=.50; Homko et al [19] [MQ], 25% in the intervention group vs 18.4% in the control group; *P*=.70). In addition, Raman et al [20] (HQ) reported an RR of 1.41 (95% CI 0.76-2.64; 228 patients) with very LQ evidence.

*Macrosomia (ie, birth weight of ≥4000 g) (4 studies)*. Overall, only 1 RCT [16] (LQ) reported a lower rate of macrosomia in the intervention group, whereas the other studies indicated either slightly lower rates in the control group or similar rates (Rasekaba et al [8] [MQ]; *P*<.05; Rasekaba et al [15] [MQ]; *P*>.99; Lemelin et al [16] [LQ]; *P*>.99; Given et al [14] [MQ]; significant statistics not reported).

*Birth weight (6 studies)*. In 2 of 3 studies with “asynchronous communication,” birth weight was slightly lower in the intervention group (Rasekaba et al [15] [MQ]; *P*>.99; Lemelin et al [16] [LQ]; *P*=.61), whereas in the 2 studies with “asynchronous and real-time communication,” birth weight was rather higher in the intervention group than in the control group (Homko et al. 2012 [19] [MQ]; *P*=.30; Given et al [14] [MQ]; significant statistics not reported).

*Respiratory distress syndrome (3 studies)*. Primarily, positive effects were obtained through telemetry. Homko et al [19] and Given et al [14] noted that GDM occurred less in the intervention groups (Homko et al [19] [MQ], 5.6% in the intervention group vs 13.2% in the control group; *P*=.40; Given et al [14] [MQ], 4.0% in the intervention group vs 15.0% in the control group; *P* value not reported).

*Shoulder dystocia (3 studies)*. Pérez-Ferre et al [13] (HQ) noted positive effects in favor of the treatment group (*P*=.50), whereas Lemelin et al [16] (LQ) reported 0.0% of positive effects in the control group and 2.5% in the intervention group (*P*=.25). In the study by Given et al [14] (MQ), no cases were detected in either group.

*Admission neonatal intensive care unit (2 studies)*. The results displayed that distinctly fewer neonates had to be admitted to the intensive care unit in the intervention group than in the control group (Given et al [14] [MQ], 36% in the treatment group vs 45% in the control group; significant statistics not reported; Homko et al [19] [MQ], 11.0% in the intervention group vs 18.4% in the control group; *P*=.60).

#### Treatment Management Outcomes

*Time saving and cost (n=4 studies)*. Primarily, telemetric interventions were explicitly associated with both time and cost savings. For example, Caballero-Ruiz et al [17] (MQ) demonstrated a significantly shorter visit duration in the intervention group (6.752 minutes vs 15.000 minutes; *P*<.01) and Lemelin et al [16] (LQ) calculated significant cost savings of 16% (Can $167.75 per patient; *P*=.003).

#### Types of Intervention

Overall, the number of studies identified was very small, and there was usually not a sufficient number of studies in both outcome classifications to be able to compare them appropriately. With the outcomes “preterm delivery,” “large for gestational age,” and “birth weight,” a direct comparison of the intervention types was possible. With regard to the outcomes LGA and birth weight, positive tendencies in favor of telemetry were observed in the studies with “asynchronous communication.” The number of preterm deliveries was lower in interventions with “asynchronous and real-time communication.”

## Discussion

### Principal Findings

In general, telemetric therapy clearly improved glycemic control, decreased the number of scheduled and unscheduled visits effectively, and some fetal and neonatal short-term outcomes indicated improved tendencies in favor of telemetry.

The findings indicate that telemetric therapy clearly improves glycemic control and effectively reduces HbA_1c_ values in women with GDM, as revealed through MQ studies. In an MQ study by Rasekaba et al [15], patients with telemetric support required significantly less insulin titrations and were therefore probably more closely metabolically adapted. They also required substantially less insulin dose_max_ units (7 units less than control subjects). Given the impact of insulin on early childhood development, this is a major finding. Telemetry also proved to be a significant predictor of better glycemic control (hazard ratio 1.71, 95% CI 1.11-2.65; *P*=.02). Furthermore, glycemic control was achieved significantly faster (4.3 weeks vs 7.6 weeks) through telemetric support, which favors a lower complication rate in the mother and child.

In addition, telemetric-supported therapy markedly reduced scheduled and unscheduled clinic visits significantly in 3 HQ, 2 MQ, and 1 LQ studies. The reduction in face-to-face clinic visits can be particularly advantageous for employed pregnant women. Less unscheduled consultations in the treatment group indicate that these women could feel secure and be better managed. This observation is reflected by the outcomes of high satisfaction of women with the telemetric applications. Despite the small sample sizes, less unscheduled consultations in insulin-treated patients indicates that this special subgroup, which usually needs closer monitoring, was probably less afraid of hypoglycemia and probably felt more secure with the treatment. However, positive tendencies were also observed with respect to higher compliance through telemetry, as revealed through MQ studies.

Early strategies are required to enhance the clinical management of GDM effectively because GDM can contribute to “programming errors” and to long-term consequences for the child. Our results indicate that telemetry, as a supportive therapy, clearly improves therapeutic safety and glycemic control among women with GDM and thus leads to a positive impact on transgenerational programming (“fetal programming”). Therefore, telemetric approaches effectively improve the clinical management of GDM and therefore contribute to a reduction in “programming errors” for the child. Unfortunately, no findings on long-term outcomes, including these diseases and other consequences, are available.

Raman et al [20] reported that the included RCTs for risk ratio calculation for these outcomes had a very low quality. Furthermore, with respect to the caesarean section rate, few MQ studies showed positive tendencies in favor of the intervention group, while more studies reported a lower rate in the control groups. This might be explained by the fact that closer supervision of the intervention group during telemetry could lead to immediate medical intervention if necessary; for example, a caesarean section. In contrast, in the less closely monitored control group, reactions may be less rapid and therefore caesarean section rates may be lower.

Regarding the fetal short-term outcomes, loss of fetal well-being, and intrauterine death, minor positive tendencies in favor of telemetric support were reported in 1 HQ and 1 MQ studies. However, owing to very small sample sizes, these outcomes should be investigated further.

Regarding the neonatal short-term outcome LGA, positive tendencies in favor of telemetric interventions were reported in the HQ study with “asynchronous communication.” The birth weight tended to be lower in telemetric interventions with “asynchronous communication” (1 HQ, 1 MQ, and 1 LQ studies). The number of preterm deliveries (<37 weeks of gestation) was clearly lower in interventions with combined “asynchronous and real-time communication,” as revealed in MQ studies. Other neonatal complications were markedly lesser with telemetric support, such as respiratory distress syndrome and the admission of neonates to the intensive care unit, but only in individual MQ studies with small sample sizes.

Additionally, telemetric-supported therapy seems to be cost-effective and time-saving. Since there are only a few investigations (MQ and LQ) in this regard, further studies are needed to assess the economic impact.

In general, only a few studies were available for our comparative analysis of the types of intervention. Based on the studies we included, a clear improvement in clinical effectiveness was observed through telemetric-supported interventions. With regard to the outcomes LGA and birth weight, positive tendencies were observed upon using “asynchronous communication” and with respect to preterm deliveries, there was a clearly positive effect of using “combined communication” (“asynchronous and real-time”). Further comparative studies are urgently required, since the type of telemetric intervention might also influence interventional effects and clinical effectiveness. Therefore, telemetric interventions have to be analyzed differently in accordance with their various technological methodologies.

### Strengths and Limitations

One of the strengths of this systematic meta-review is our development and application of a unique classification system for the telemetric technologies used in diabetes management. We allocate an in-depth review by incorporating different study designs. Furthermore, we have considered a wide range of outcomes in our systematic meta-review. With our differentiated and detailed analysis based on outcomes, intervention types, study quality, and sample sizes, our study provides substantiated findings.

Given the limitations, different national guidelines worldwide as well as the resulting different threshold values for the diagnosis of GDM affect the comparability of these studies. With different screening methods and definitions of GDM, participants may not be precisely comparable. Although telemetric interventions are critical among pregnant women, only a small number of studies have focused on this field.

Furthermore, HbA_1c_ is of limited value as a metric for evaluating glucose control in GDM, but the studies included in this systematic meta-review focused on HbA_1c_ levels and did not provide sufficient information on mean postprandial and fasting glycemia.

### Comparison With Prior Studies

Our results regarding the reduction in HbA_1c_ values ​​through telemetric-supported therapy are concurrent with those of Ming et al [9], who analyzed telemedicine technologies for diabetes mellitus in pregnancy, examined the subgroup of women with GDM, and reported a significant reduction in HbA_1c_ levels with an MD of –1.14% (95% CI 0.25-0.04). Rasekaba et al [8] investigated 3 RCTs and also concluded that glycemic control indicated an improving trend in favor of telemetric interventions. The authors outlined the advantages of telemetric systems in the reduction of face-to-face and unscheduled clinic consultations, which is consistent with our results. With respect to maternal and fetal or neonatal complications, Rasebaka et al [8] (3 studies) and Raman et al [20] (5 relevant studies) indicated in their meta-analyses that the complication rates in telemetric interventions and control groups (usual care) were similar. However, in our systematic meta-review, we could identify minor positive trends with regard to fetal and neonatal outcomes.

### Conclusions

In conclusion, our findings indicate clear improvement in glycemic control, particularly an improvement in HbA_1c_ values, through telemetric therapy. Rasekaba et al [15], reported that significantly less insulin titrations were required, glycemic control was achieved significantly earlier, and telemetry was determined as a significant predictor for a better glycemic control. Since the needs for scheduled and unscheduled clinic visits obviously declined significantly, women probably felt more secure and supervised during telemetric-supported GDM therapy. In addition, the women seemed to be highly satisfied with the telemetric therapy. This systematic meta-review shows that telemetric-supported therapy markedly improves glycemic control among women with GDM and thus leads to a positive impact on transgenerational programming (“fetal programming”). Regarding fetal and neonatal short-term outcomes, some improving tendencies in favor of telemetric interventions were detected. These positive effects could only be achieved through telemetry itself, which reinforces this new digital approach in the treatment of GDM.

Telemetric interventions tend to save costs and time, but further studies are needed to determine the economic impact of this digital approach. We could not identify any publications for our categories of “real-time video communication” and “real-time audio communication.” Since the type of intervention, namely the technologies used, might also influence clinical effectiveness, the effects of different intervention types should be investigated in more detail in future studies. Furthermore, studies are still needed to consider the long-term outcomes of these interventions.

## Appendix

Multimedia Appendix 1 Search terms and strategy in the databases.

Multimedia Appendix 2 PRISMA flowchart (adapted from Moher et al 2009).

Multimedia Appendix 3 Characteristics of the included studies (n=11).

Multimedia Appendix 4 Quality assessments.

Multimedia Appendix 5 Effects on maternal, fetal and neonatal outcomes.

## Abbreviations

GDM: gestational diabetes mellitus
HbA_1c_: glycated hemoglobin A_1c_
HQ: high quality
LGA: large for gestational age
MA: meta-analysis
MD: mean difference
MQ: moderate quality
PRISMA: Preferred Reporting Items for Systematic Reviews and Meta-Analyses
RCT: randomized controlled trial
RR: risk ratio
SR: systematic review
