# Supplementary material for: Effects of Telemetric Interventions on Maternal and Fetal or Neonatal Outcomes in Gestational Diabetes: Systematic Meta-Review
Source: JMIR Diabetes. 2021 Aug 27;6(3):e24284. doi: 10.2196/24284 (PMC8433929; doi:10.2196/24284)
Supplement: Multimedia Appendix 1 [file diabetes_v6i3e24284_app1.docx]

**Search terms and strategy in the databases.**

|  | PUBMED | EMBASE | COCHRANE | CINAHL | WEB OF SCIENCE  CORE COLLECTION |
| --- | --- | --- | --- | --- | --- |
| KEYWORDS | diabetes mellitus, gestational diabetes, telemetry, telemedicine, telemonitoring | diabetes mellitus, pregnancy diabetes mellitus, gestational diabetes, telemetry, telemedicine, telemonitoring | diabetes mellitus, gestational diabetes, telemetry, telemedicine, telemonitoring | diabetes mellitus, gestational diabetes, telemetry, telemedicine, telemonitoring | diabetes mellitus, gestational diabetes, telemetry, telemedicine, telemonitoring |
| FILTERS | clinical trial, meta-analysis, randomized controlled trial, systematic review; publication date from 2008/01/01 to 2020/12/31; English; German | clinical trial, meta-analysis, randomized controlled trial, systematic review; 2008­­­-2020; English; German | cochrane reviews, trials and clinical answers; from January 2008 to April 2020; English; German | clinical trial, meta-analysis, randomized controlled trial, systematic review; 2008/01/01-2020/04/02; English, German | 2008-2020; English; German |
| STRATEGY | ((((("diabetes mellitus"[Title/Abstract]) OR "Diabetes Mellitus"[MeSH Terms]) OR "gestational diabetes"[Title/Abstract]) OR diabetes, gestational[MeSH Terms])) AND (((((("Telemetry"[Mesh]) OR "Telemedicine"[Mesh]) OR telemonitoring[Title/Abstract]) OR "telemetry"[Title/Abstract]) OR "telemedicine"[Title/Abstract])) | ((‘diabetes mellitus’/exp OR ‘pregnancy diabetes mellitus’/exp OR ‘diabetes mellitus’:ab,ti OR ‘gestational diabetes’: ab,ti) AND (‘telemetry’/exp OR ‘telemedicine’/exp OR ‘telemonitoring/exp OR ‘telemedicine:ab,ti OR ‘telemonitoring:ab,ti OR ‘telemetry’:ab,ti)) | ((MeSH [Diabetes Mellitus] OR MeSH [Diabetes, Gestational] OR “diabetes mellitus”:ti,ab OR “gestatinal diabetes”:ti,ab) AND (MeSH [Telemedicine] OR MeSH [Telemetry] OR “telemedicine”:ti,ab OR “telemetry”:ti,ab OR “telemonitoring”:ti,ab)) | ((MH “diabetes mellitus” OR TI “diabetes mellitus” OR AB “diabetes mellitus” OR TI “gestational diabetes” OR AB “gestational diabetes”) AND (MH telemedicine OR MH telemetry OR TI telemetry OR AB telemetry OR TI telemedicine OR AB telemedicine OR TI telemoritoring OR AB telemonitoring)) | ((TOPIC “diabetes mellitus” OR TOPIC “gestational diabetes”) AND (TOPIC telemetry OR TOPIC telemedicine OR TOPIC telemonitoring)) |
