# Supplementary material for: Effects of Telemetric Interventions on Maternal and Fetal or Neonatal Outcomes in Gestational Diabetes: Systematic Meta-Review
Source: JMIR Diabetes. 2021 Aug 27;6(3):e24284. doi: 10.2196/24284 (PMC8433929; doi:10.2196/24284)
Supplement: Multimedia Appendix 2 [file diabetes_v6i3e24284_app2.docx]

**PRISMA flowchart (adapted from Moher et al. 2009).**

Records identified through database searching (including GDM, T1DM, and T2DM)

(n=1647)

n=450 (MEDLINE via PubMed)

n=406 (EMBASE)

n=81 (CINAHL)

n=368 (Cochrane Library)

n=346 (Web of Science Core Collection)

April 2020

## Identification

Publications not fulfilling search criteria based on
title/abstract
(n=875)

Titles/abstracts screened after duplicates removed (n=1116)

## Screening

Studies excluded (n=72)

Pooled Data (n=4)

Not diabetes therapy
(n=2)

Study design (n=3)

Method description only (n=1)

App–based (n=60)

No access (n=2)

Full-text studies assessed for eligibility
(n=241)

Studies included after
assessing full-texts
(n=169)

## Eligibility

Total studies (n=184)

*GDM (n=11)
T1DM (n=23)*

*T2DM (n=99)*

*T1DM/T2DM (n=51)*

Scrutiny of reference lists to identify further studies
(n=15)

## Included

GDM studies included in final synthesis (n=11)
