# Supplementary material for: Effects of Telemetric Interventions on Maternal and Fetal or Neonatal Outcomes in Gestational Diabetes: Systematic Meta-Review
Source: JMIR Diabetes. 2021 Aug 27;6(3):e24284. doi: 10.2196/24284 (PMC8433929; doi:10.2196/24284)
Supplement: Multimedia Appendix 3 [file diabetes_v6i3e24284_app3.docx]

**Characteristics of the included studies (n=11).**

| Study | | Topic | Design | Participants/  included studies | Intervention/ (Control) | Duration (Months) | Outcomes | Key results | Author’s conclusions |  |
| --- | --- | --- | --- | --- | --- | --- | --- | --- | --- | --- |
|  | **Systematic reviews and meta-analyses (n=4)** | | | | | | | | |  |
| (Rasekaba et al. 2015)  Australia | | Telemedicine interventions for gestational diabetes  mellitus | SR & MA | RCTs (n=3)  Patients (n=243) | Web–based; app–based and SMS communication; interactive voice response and asynchronous messaging | - | Face-to-face visits  Unscheduled visits  Fasting blood glucose  2-h postprandial (breakfast, dinner and lunch)  HbA_1c_  Birth weight  Macrosomia  Caesarean section rate  Quality of life | - Telemedicine associated with significantly fewer unscheduled clinic visits (*P=*0.033 and insulin treated subgroup *P=*0.0001) - Lower face-to-face visits *P=*0.733 and insulin treated subgroup *P=*0.002 - No statistically significant differences in glycaemic control, birth weight, macrosomia, caesarean deliveries - Caesarean: OR 0.48 (95% CI: 0.10 to 2.35) - Sub-domains of diabetes self-efficacy: managing the psychosocial aspects of diabetes (*P=*0.039) and Setting and Achieving Diabetes Goals (*P=*0.036)   MA showed small effect sizes, not significant:   - HbA_1c_: MD -0.18% (95% CI -0.50 to 0.14) *P=*0.27 - 1-h and 2-h post-prandial BGL: MD -0.02% (95% CI -0.36 to 0.32) *P=*0.39 | Positive: Advantage may lie in the ability to deliver support; monitoring remotely for the convenience; reducing face-to-face and unscheduled consultations |  |
| (Raman et al. 2017)  - | | Different methods and settings for glucose monitoring for gestational diabetes | Review quali-tative and quanti-tative analysis | RCTs (n=11); of which n=5 are relevant (telemetry)  Patients (n=478) | Data transmission: internet–based, cellular phone (app–based), telemedicine-hub; weekly in all interventions  Feedback: telephone voice messages, cellular telephone text messages, telephone calls, web–based | - | Pre-eclampsia  Ceasarean section  Induction of labour  Large-for-gestational age  Death/serious morbidity composite  Neonatal hypoglycaemia | No clear differences for mother:   - Pre-eclampsia RR 1.49 (95% CI: 0.69 to 3.20 (n=275 patients) - Caesarean section RR 1.05 (0.72 to 1.53) (n=478) - Induction of labour RR 1.06 (0.63 to 1.77) (n=74)   No clear differences for child:   - Large-for-gestational age RR 1.41 (0.76 to 2.64) (n=228 patients) - Death or serious morbidity composite RR 1.06 (0.68 to 1.66) (n=57) - Neonatal hypoglycaemia RR 1.14 (0.48 to 2.72) (n=198) | Unclear which methods/settings are best |  |
| (Ming et al. 2016)  Europe & North America | | Telemedicine technologies for diabetes in pregnancy | SR & MA | Total RCTs (n=7)  Only GDM RCTs (n=4)  Only GDM patients (n=416) | Technologies: modem transmission; web–based; telephone system; SMS; telemedicine hub | - | HbA_1c_ | - Mean HbA_1c_ telemetry 5.22% (SD 0.70) standard care 5.37% (SD 0.61); MD -1.14% (95% CI -0.25 to 0.04) *P=*0.01   *(Other Outcomes pooled results with other types of diabetes)* | Insufficient, but no evidence of damage; 7 trials included were all small and assessed different technologies (and different types of diabetes) |  |
| (Fantinelli et al. 2019)  US, Switzerland, Spain, Australia, Europe, Korea | | Assessment of psychological  dimensions in telemedicine care | SR of quali-tative and quanti-tative studies | n=13 quantitative and qualitative studies  Patients (n=982) | E.g. Internet-based/ app-based/ web-based systems, text messaging, telemedicine hub, phone call | - | Psychological dimensions:  Self-efficacy  Satisfaction  Compliance | - In one study, IG revealed higher feelings of diabetes self-efficacy than CG (*P=*0.053) and IG needed more insulin therapy compared to CG (*P<.*05) (maybe because of short intervention period) - N=4 studies showed IG were more compliant than CG (significance not reported) - In one study, IG reported more blood values than CG (*P<.*001) - IG highly satisfied (different questionnaires) *P=*0.71, *P<.*001 (other studies without P-values reported) | Results are not sufficient to state a conclusive evaluation of positive effects of telemedicine use for GDM care |  |
|  | **“Asynchronous interventions” (n=4)** | | | | | | | | | |
| (Rasekaba et al. 2018)  Australia | | Using technology to support care in gestational diabetes mellitus (TeleGDM) | RCT | Baseline and analyzed:  IG (n=61) CG (n=34)  Up to 35 weeks  Gestation | IG: usual care + web–based portal for data transmission (daily) and communication; feedback when needed  CG: face-to-face appointments every one to two weeks; paper diary | 2,5 | Face-to-face appointments  Glycaemic control  Insulin Dose_max_  Insulin titrations  Ceasarean delivery  Biparietal diameter  Head circumference  Abdominal circumference  Femur length  Birth weight  Macrosomia  Costs | - No differences between groups face-to-face appointments median (IQR) IG=8(7) CG=8(6) *P=*0.843, - IG reached optimum glycaemic control (maximum dose of insulin) quicker 4.3 (SD 4.2) weeks vs. 7.6 (SD 4.5) weeks *P=*0.0001 - Telemedicine significant predictor of better glycaemic control HR=1.71 (95% CI: 1.11 to 2.65, *P=*0.015) - Insulin Dose_max_ not significantly different IG=22(SD 17) units vs. control=29(25) units, *P=*0.24 - IG required significantly fewer insulin titrations median (IQR)=4(13) vs. 13(25) *P=*0.04 - Incidence ceasarean delivery higher in IG 46% (95% CI: 33 to 59) vs. 32% (95% CI: 17 to 51) *P=*0.20   3^rd^ trimester foetal outcomes:   - Biparietal diameter (cm) IG 8.4 (0.6) vs. CG 8.2 (0.7), *P=*0.17 - Head circumference (cm) IG 30.6 (1.9) vs. 30.0 (2.3), *P=*0.15 - Abdominal circumference (cm) IG 29.6 (2.7) vs. 29.4 (3.4), *P=*0.77 - Femur length (cm) IG 6.3 (0.6) vs. 6.3 (0.6), *P=*0.51 - Birth weight (g) IG 3311 (455) vs. 3275 (384), *P=*0.69 - Macrosomia (%) IG 4.9 (1.0 to 13.7) vs. 2.9 (0.1 to 15.3), *P=*1.00   Service provider costs:   - IG vs. CG mean(SD): face-to-face appointments AU$2506.23(1539.09) vs. 2305.88(1163.81), *P=*0.71, planned appointments AU$1735.08(1554.63) vs. AU$1869.41(1175.31), *P=*0.23 and unplanned AU$968.52(1048.38) vs. AU$675.29(972.68), *P=*0.05 | Intervention may be associated with fewer insulin dose titrations and patients achieved glycaemic control quicker |  |
| (Perez-Ferre et al. 2010a)  Spain | | Telemedicine system –based on internet and short  message service | RCT, pilot | Baseline:  IG (n=50) CG (n=50)  24 to 40 weeks gestation  Analyzed:  IG (n=49)  CG (n=48) | IG: Telemedicine (internet and app–based via SMS)  CG: face-to-face outpatient clinic visits | Ca. 4 | Face-to-face visits  Unscheduled visits  Gestational weeks at insulinitation | - Face-to-face visits IG 3.98±0.99 vs. CG 4.34±1.73, *P=*0.733 - Unscheduled visits IG 0.38±0.68 vs. CG 1±1.35, *P=*0.033   Insulin-treated patients:   - Gestational weeks at insulinitation IG 27.73±3.13 vs. CG 28.22±3.80, *P=*0.727 - Face-to-face visits IG 4.25±0.93 vs. CG 6.22±1.48, *P=*0.002 - Unscheduled visits IG 0.50±0.73 vs. CG 2.89±1.05, *P=*0.0001 | Telemedicine as useful tool as complement to conventional face-to-face monitoring |  |
| (Perez-Ferre et al. 2010b)  Spain | | Outcomes of gestational diabetes mellitus after a telecare approach are not inferior to outpatient clinic visits | RCT | Baseline:  IG (n=50) CG (n=50)  24 to 40 weeks gestation  Analyzed:  IG (n=49)  CG (n=48) | IG: Telemedicine (internet and app–based via SMS)  CG: face-to-face outpatient clinic visits | Ca. 4 | Pregnancy induced hypertension  Normal vaginal birth  Caesarean section  Birth weight  Large for gestational age  Loss of fetal wellbeing  Outpatient clinic visits  Weight gain  HbA_1c_  Contacts and time  Hypoglycemia  Hypocalcemia  Poliglobulia  Small-for-gestational age  Preterm birth  Umbilical cord pathology  Abruptio placentae  Blood pressure  Albumin-to-creatinine ratio  Time saving | - Pregnany induced hypertension IG 4.1% vs. CG 0% *P=*0.501 - Normal vaginal birth IG 40.8% vs. 54.2% *P<.*68 - Instrumental vaginal birth IG 10.4% vs. 20.4%, *P=*0.427 - Caesarean section IG 34.7% vs. 25% *P=*0.427 - Birth weight IG 3308.2g±488.8 vs. 3370.6g±479.1, *P=*0.385 - Large for gestational age IG 6.1% vs. 8.3%, *P=*0.500 - Loss of fetal wellbeing IG 6.1% vs. 10.4%, *P=*0.500 - Shoulder dystocia IG 0.0% vs. 2.1%, *P=*0.500 - 62% reduction outpatient clinic visits in IG and 82% in the case of insulin-treated women, *P<.*03 - Hypoglycemia IG 0% vs. 0%, *P=*0.500 - Hypocalcemia IG 0% vs. 0%, *P=*0.500 - Poliglobulia IG 0% vs. 0%, *P=*0.500 - Small-for-gestational age IG 0% vs. 0%, *P=*0.500 - Preterm birth (<37 weeks) IG 2.1% vs. 2.0%, *P=*0.500 - Umbilical cord pathology IG 4.2% vs. 2.0%, *P=*0.500 - Abruptio placentae IG 2.1% vs. 0%, *P=*0.500 - All women HbA_1c_ <5.8% - IG had more contacts (15.05 vs. 9.11) taking up less time (3.8 vs. 4.6 hours; *P<.*001)   Maternal metabolic parameters Visit 1 (before 28 weeks) and visit 4 (39-40 weeks):   - Systolic blood pressure (mmHg) visit 1 (CG 122.0±16.8 IG 122.3±12.5) vs. visit 4 (CG 120.8±14.8 IG 122.9±10.8) - Diastolic blood pressure (mmHg) visit 1 (CG 71.5±8.6 IG 72.6±9.5) vs. visit 4 (CG 72.1±8.0 IG 76.8±10.6) - HbA_1c_ (%) visit 1 (CG 5.2±0.4 IG 5.0±0.4) vs. visit 4 (CG 5.4±0.4 IG 5.3±0.4) - Albumin-to-creatinine ratio (mg/g) visit 1 (CG 6.8±4.9 IG 10.1±14.4) vs. visit 4 (CG 5.1±2.9 IG 7.7±5.3) - Weight gain (kg) visit 1-4 (CG 6.446±4.988 IG 5.822±3.950), *P>.*05 | Neutral:  System significantly reduces the need for outpatient clinic visits and achieves similar pregnancy, delivery, and newborn outcomes |  |
| (Lemelin et al. 2020)  Canada | | Demonstrated cost-effectiveness of a telehomecare  program | Non-rando-mized con-trolled clinical trial | Baseline and analyzed: IG (n=80) CG (n=81) | IG: web–based system (Telehomecare “THCa”) for data transmission and communication; feedback at least every 2 weeks  CG: usual care; not adequately described  Both: no predetermined number of visits in clinic | IG: 7 CG: 8 | Medical visits  Obstetrical emergency visits  Satisfaction  Cost  Nursing interventions  Ceasarean delivery  Assisted vaginal delivery  (Pre-)Eclampsia  Gestational hypertension  Body weight  Preterm delivery  Macrosomia  Large for gestational age  Neonatal hypoglycemia  Hyperbilirubinemia  Respiratory distress  Shoulder dystocia  Premature rupture of membranes | - Decrease medical visits by 56% in IG, *P<.*001 (IG 1.5 visits vs. CG 3.3 visits) - Patients with ≥1 visit to the obstetrical emergency (%) IG 26.3 vs. CG 25.9, *P=*0.962 - Absenteeism at work (half-day±SD) IG 2.0±2.3 vs. CG 3.0±3.0, *P=*0.014 - Global satisfaction similar *P=*0.128 and satisfaction with educational support significantly increased in IG *P=*0.028) - Significant cost savings of 16% (167.75CAN$ per patient), *P=*0.003 - Increase in nursing interventions by about 10-fold (10.3 vs. 0.9, *P<.*001) in IG (by e-mails and phone calls) - Ceasarean delivery IG 18.8% vs. CG 32.1%, *P=*0.070 - Assisted vaginal delivery 17.5% vs. 12.3%, *P=*0.359 - (Pre-)Eclampsia 0% vs. 0% - Gestational hypertension 1.3% vs. 2.5%, *P=*0.230 - Body weight 3263g±336 vs. 3292g±391, *P=*0.608 - Preterm delivery <37 weeks 3.8 vs. 0.0, *P=*0.079 - Macrosomia ≥4000g 1.3% vs. 2.5%, *P=*1.000 - Large for gestational age 2.5% vs. 3.7%, *P=*1.000 - Neonatal hypoglycemia 20.0% vs. 16.0%, *P=*0.545 - Hyperbilirubinemia treated by phototherapy 5.0% vs. 8.6%, *P=*0.534 - Respiratory distress syndrome 1.3% vs. 1.2%, *P=*1.000 - Shoulder dystocia 2.5% vs. 0.0%, *P=*0.245 - Premature rupture of membranes IG=11.3% vs. 14.8%, *P=*0.502 | Positive:  THCa monitoring significantly decreases medical visits and direct costs without compromising pregnancy outcomes, quality of care, or patient satisfaction |  |
|  | **“Combined interventions: real-time and asynchronous communication” (n=3)** | | | | | | | | |  |
| (Given et al. 2015)  Northern Ireland, Republic of Ireland | | Tele-Mum:  feasibility study  exploring the potential for telemedicine in the diabetes care | RCT | Baseline:  IG (n=24)  CG (n=26)  Analyzed:  IG (n=21)  CG (n=26) | IG: usual care + telemedicine-hub (landline, mobile phone, website); weekly feedback  CG: usual care; face-to-face clinic visits at least every 2 weeks; SMBG | NA | Advantages and desadvantages using telemedicine system  Satisfaction  HbA_1c_  Appointments attended  Preeclampsia/ pregnancy induced hypertension  Cesarean delivery  Premature  Macrosomia  Neonatal hypoglycemia  Respiratory distress  Shoulder dystocia  Intrauterine death  Birth weight | - Satisfaction rates IG 91% and CG 85% - Easy to use and reassuring, would recommend - HbA_1c_ at 36 weeks gestation IG 34.04mmol/mol ± 3.23 vs. 33.84±2.88 - Appointments attended IG 97.8%±6.1 vs. 92.6±18.2 - Pre-eclampsia/pregnancy induced hypertension IG 0.0% vs. 3.9% - Cesarean delivery IG 47.6% vs. 38.5% - Premature <37 weeks of gestation IG 0% vs. 8% - Macrosomia IG 28.6% vs. 8% - Neonatal hypoglycemia (<2.6 mmol/L) IG 20% vs. 25% - Respiratory distress IG 4.0% vs. 15.0% - Shoulder dystocia IG 0.0% vs. 0.0% - Intrauterine death IG 0.0% vs. 3.9% - Birth weight (g) IG 3557±599 vs. 3272±443 - Admitted to neonatal intensive care unit IG 36% vs. 45% - Jaundice 50% vs. 47.1%   *(no P-values reported)* | Telemedicine can replace face-to-face appointments and help meet the growing demand for diabetes services as more and more women are diagnosed with GDM |  |
| (Homko et al. 2012)  US | | Impact of a telemedicine system with automated  reminders | RCT | Baseline:  IG (n=40)  CG (n=40)  Analyzed:  IG (n=36)  CG (n=38) | IG: weekly data transmission and feedback via internet–based system; interactive voice response telephone communication, educational messages  CG: logbook reviewed at clinic visits  Both groups: clinic visits every 2 weeks and weekly from 36 weeks gestations | Ca. 2 | Fasting blood sugar  Mean blood glucose  Glucose breakfast, lunch, dinner  Ceasarean delivery  Pre-eclampsia  Birth weight  Premature rupture of membranes  Chorioamnionitis  Large for gestational age  Neonatal hypoglycemia  Preterm delivery  Respiratory distress syndrome  Jaundice/hyperbilirubinemia  Apgar score | - Fasting blood sugar IG 91.5mg/dL±10.5 vs. CG 94.3±10.5, *P=*0.26 - Glucose breakfast IG 108.1mg/dL±17.0 vs. 110.9±18.5, *P=*0.50 - Glucose lunch IG 110.6±17.2 vs. 111.1±18.5, *P=*0.90 - Glucose dinner IG 119.9±17.3 vs. 117.6±22.0, *P=*0.64 - Mean glucose IG 107.4±12.9 vs. 109.7±16.5, *P=*0.44 - Ceasarean delivery IG 36% vs. 50%, *P=*0.3 - Pre-eclampsia IG 8% vs. 5%, *P=*0.7 - Premature rupture of membranes IG 5.5% vs. 0%, *P=*0.2 - Chorioamnionitis IG 2.8% vs. 2.6%, *P=*1.0 - Birth weight IG 3372±469 vs. 3249±611, *P=*0.3 - Large for gestational age IG 25% vs. 18.4%, *P=*0.7 - Neonatal hypoglycemia IG 11% vs. 10.5%, *P=*1.0 - Preterm delivery IG 5.6% vs. 13.2%, *P=*0.4 - Jaundice/hyperbilirubinemia IG 2.8% vs. 5%, *P=*1.0 - Respiratory distress syndrome IG 5.6% vs. 13.2%, *P=*0.4 - Two-thirds of women in IG achieved mean glucose level of < 110 mg/dL - Apgar score 1 min IG 7.6±2.0 vs. 8.1±1.0, *P=*0.2 Apgar score 5 min IG 8.9±0.5 vs. 8.9±0.3, *P=*0.8 - Intensive care unit admissions IG 11% vs. 18.4%, *P=*0.6 | No impact on pregnancy outcomes; potential  may be ability to  increase efficiency while maintaining quality of care |  |
| (Caballero-Ruiz et al. 2017)  Spain | | Web–based clinical decision support system for  gestational diabetes | RCT | Baseline:  IG (n=60)  CG (n=30)  Analyzed:  IG (n=60)  CG (n=29)  33 weeks of gestation or less at entry | IG: web–based telemedicine platform: “Sinedie” (Smart and educational system for gestational diabetes); instant feedback after upload (every 3 days) from endocrinologist; includes education; telephone calls  IG: face-to-face clinic visits; not adequately described | 17 | Face-to-face visits  Workload/Time saving  Visit duration  Number BG measurements  Satisfaction | - Reduced face-to-face visits per patient by 88.56% (3.207±2.846 in CG vs. 0.367±0.901 in IG), *P<.*01 - Time spent evaluating patients by doctors was reduced by 27.389% (no p-value reported) - Visit duration CG 15.000 min vs. 6.752 min IG (*P<.*01) - Number BG measurements per patient (*P>.*05) IG 147.017 vs. CG 141.562 - Patients highly satisfied (no P-value reported) | “Sinedie” provides safe advice on therapy adjustments and reduces the workload of doctors |  |

BGL = blood glucose level; CC = control group; CI = conficence interval; HbA_1c_ = hemoglobin A_1c_; IG = intervention group; IQR = interquartile range; MA = meta-analysis; MD = mean difference; NA = not available; OR = odds ratio; RCT = randomized controlled trial; RR = relative risk; HR = hazard ratio, SD = standard deviation; SR = systematic review
