# Supplementary material for: Effects of Telemetric Interventions on Maternal and Fetal or Neonatal Outcomes in Gestational Diabetes: Systematic Meta-Review
Source: JMIR Diabetes. 2021 Aug 27;6(3):e24284. doi: 10.2196/24284 (PMC8433929; doi:10.2196/24284)
Supplement: Multimedia Appendix 4 [file diabetes_v6i3e24284_app4.docx]

**Quality assessments.**

Table A-1: Quality assessment of systematic reviews and meta-analyzes (n=4) (AMSTAR 2: A MeaSurement Tool to Assess systematic Reviews).

|  | 1 | 2 | 3 | 4 | 5 | 6 | 7 | 8 | 9 | 10 | 11 | 12 | 13 | 14 | 15 | 16 |  |
| --- | --- | --- | --- | --- | --- | --- | --- | --- | --- | --- | --- | --- | --- | --- | --- | --- | --- |
| Question/ Study | PICO | Priori Design | Selection study designs | Compre-hensive search strategy | Study selection in duplicate | Data extrac-tion in duplicate | List exclu-ded studies | Des-cription studies | Assessment RoB | Sorces of funding | Statis-tical combination (MA) | Impact of RoB on results (MA) | Account for RoB when inter-preting | Hetero-geneity | Publi-cation bias (MA) | Conflict of interest |  |
| High quality (n=1) | | | | | | | | | | | | | | | | | |
| Raman et al. 2017 | + | + | + | + | + | + | o | + | + | + | + | + | + | + | + | + |  |
| Moderate quality (n=3) | | | | | | | | | | | | | | | | | |
| Fantinelli et al. 2019 | + | + | + | o | - | - | - | + | + | - | / | / | + | + | / | + |  |
| Ming et al. 2016 | + | + | - | o | + | + | - | + | + | - | + | + | + | + | + | + |  |
| Rasekaba et al. 2015 | + | + | - | o | + | + | - | + | + | - | + | + | + | + | - | + |  |

+ = criterion met, - = criterion not met, o = criterion partially met, / = not applicable, MA = meta-analysis, RoB = risk of bias

Table A-2: Quality assessment of trials (n=7) (EPHPP: Effective Public Health Practice Project).

| Question/ Trial | A Seletion Bias (Q1) | A Selection Bias (Q2) | A SCORE | B Study Design | B SCORE | C  Confoun-ders (Q1) | C  Confoun-ders (Q2) | C SCORE | D Blinding (Q1) | D Blinding (Q2) | D SCORE | E  Data collection (Q1) | E  Data Collection (Q2) | E SCORE | F Withdrawals and Drop-outs (Q1) | F Withdrawals and Drop-outs (Q2) | F SCORE | GLOBAL RATING |
| --- | --- | --- | --- | --- | --- | --- | --- | --- | --- | --- | --- | --- | --- | --- | --- | --- | --- | --- |
| Perez-Ferre et al. 2010a | 4 | 1 | ** | 1 | *** | 2 | / | *** | 3 | 3 | ** | 1 | 1 | *** | 1 | 1 | *** | **Strong** |
| Perez-Ferre et al. 2010b | 4 | 1 | ** | 1 | *** | 2 | / | *** | 3 | 3 | ** | 1 | 1 | *** | 1 | 1 | *** | **Strong** |
| Rasekaba et al. 2018 | 4 | 1 | ** | 1 | *** | 2 | / | *** | 1 | 1 | * | 1 | 1 | *** | 1 | 1 | *** | **Moderate** |
| Given et al. 2015 | 4 | 2 | ** | 1 | *** | 2 | / | *** | 1 | 1 | * | 1 | 1 | *** | 1 | 1 | *** | **Moderate** |
| Homko et al. 2012 | 4 | 5 | * | 1 | *** | 2 | / | *** | 3 | 3 | ** | 1 | 1 | *** | 1 | 1 | *** | **Moderate** |
| Caballero-Ruiz et al. 2017 | 4 | 5 | * | 1 | *** | 2 | / | *** | 3 | 3 | ** | 1 | 1 | *** | 1 | 1 | *** | **Moderate** |
| Lemelin et al. 2020 | 4 | 5 | * | 2 | *** | 2 | / | *** | 1 | 1 | * | 1 | 1 | *** | 1 | 1 | *** | **Weak** |

* = week, ** = moderate, *** = strong, 1-5 = response options according to EPHPP, / = not applicable
