# Supplementary material for: Effects of Telemetric Interventions on Maternal and Fetal or Neonatal Outcomes in Gestational Diabetes: Systematic Meta-Review
Source: JMIR Diabetes. 2021 Aug 27;6(3):e24284. doi: 10.2196/24284 (PMC8433929; doi:10.2196/24284)
Supplement: Multimedia Appendix 5 [file diabetes_v6i3e24284_app5.docx]

**Effects on maternal, fetal and neonatal outcomes.**

Table B-1: Effects on maternal outcomes

| **Outcome** | **Study, effects, study quality, sample size, study type/intervention type** | | | | | | | | | | | | | |
| --- | --- | --- | --- | --- | --- | --- | --- | --- | --- | --- | --- | --- | --- | --- |
| **HbA1c (% or mmol/mol)** | **Rasekaba et al. 2015**  MD -0.18% (-0.50, 0.14)² P=0.27 | | **Ming et al. 2016**  MD -1.14% (-0.25, 0.04) P=0.01 | | | | | **Perez-Ferre et al. 2010b**  Visit 1³ (CG 5.2%±0.4 IG 5.0%±0.4) vs.  Visit 4 (CG 5.4%±0.4 IG 5.3%±0.4)  All <5.8%, SNA | | | | **Given et al. 2015**  IG=34.04mmol/mol ± 3.23  CG= 33.84±2.88  (at 36 weeks), SNA | | |
|  | ●●◯ MODERATE ^1^  IG (n=81), CG (n=63) SR/MA | | ●●◯ MODERATE  IG (n=196), CG (n=220)  SR/MA | | | | | ●●● HIGH  IG (n=49), CG (n=48)  Asynchronous ^2^ | | | | ●●◯ MODERATE  IG (n=21), CG (n=26)  Asynchronous and real-time ^3^ | | |
| **Insulin dose (units) Insulin titrations (quantity) Glycemic control** | **Rasekaba et al. 2018** ●●◯ MODERATE IG (n=61), CG (n=34) Asynchronous | | | | | | | | | | | | | |
|  | Insulin dose _max_ (units)  IG=22±17, CG=29±25, P=0.24 | | Insulin titrations (quantity)  Median(IQR) IG=4(13), CG=13(25), P=0.04 | | | | | Telemetry as predictor better glycemic control HR=1.71 (1.11, 2.65), P=0.015 | | | | Weeks to achieve glycemic control (insulin dose_max_)  IG=4.3±4.2, CG=7.6±4.5, P=0.0001 | | |
| **Gestational weeks at insulinitation** | **Perez-Ferre et al. 2010a ●●●** HIGH IG (n=17), CG (n=9) Asynchronous  IG=27.73±3.13, CG=28.22±3.80, P=0.727 | | | | | | | | | | | | | |
| **Face-to-face visits  (quantity or reduction  in %)** | **Rasekaba et al. 2015**  ↓ in IG P=0.733 | **Rasekaba et al. 2018**  Median(IQR) IG=8(7),  CG=8(6), P=0.843 | | | **Perez-Ferre et al. 2010a**  IG=3.8±0.99  CG=4.34±1.73, P=0.733  Insulin-treated: IG 4.25±0.93, CG 6.22±1.48, P=0.002 | | | **Perez-Ferre et al. 2010b**  IG=62% ↓  Insulin-treated: IG=82% P<0.03 | | **Lemelin et al. 2020**  IG=56% ↓  P<0.001 | | | | **Caballero-ruiz et al. 2017**  IG=88.56% ↓  P<0.01 |
|  | ●●◯ MODERATE  IG (n=49), CG (n=48)  SR/MA | ●●◯ MODERATE  IG (n=61), CG (n=34) Asynchronous | | | **●●●** HIGH  IG (n=49), CG (n=48)  Insulin: IG (n=17), CG (n=9)  Asynchronous | | | **●●●** HIGH  IG (n=49), CG (n=48)  Asynchronous | | ●◯◯ LOW IG (n=80), CG (n=81)  Asynchronous | | | | ●●◯ MODERATE  IG (n=60), CG (n=29)  Asynchronous and real-time |
| **Unscheduled visits (quantity)** | **Rasekaba et al. 2018**  ↓ in IG P=0.033 | | | | | **Perez-Ferre et al. 2010a**  IG=0.38±0.68, CG=1±1.35  P=0.033 | | | | **Lemelin et al. 2020**  IG 26.3 CG 25.9 P=0.962 | | | | |
|  | ●●◯ MODERATE  IG (n=49), CG (n=48)  Asynchronous | | | | | **●●●** HIGH  IG (n=49), CG (n=48)  Asynchronous | | | | ●◯◯ LOW  IG (n=80), CG (n=81)  Asynchronous | | | | |
| **Patients with ≥1 visit  to the obstetrical emergency** | **Lemelin et al. 2020** ●◯◯ LOW Asynchronous  IG 2.0±2.3 , CG 3.0±3.0, P=0.014 | | | | | | | | | | | | | |
| **Compliance** | **Fantinelli et al. 2019** IG more compliant, SNA | | | | | **Homko et al. 2012**  Data transmitted: 17.4±16.9 in previous conducted study to 35.6± 32.3 in this study (with automated reminders), P<0.01 | | | | | **Caballero-Ruiz et al. 2017**  BG measurments/patient (mean/SD): IG=147.017(144.485), CG=141.562(123.717), P>0.05 | | | |
|  | ●●◯ MODERATE  n=401  SR | | | | | ●●◯ MODERATE  IG (n=36), CG (n=38)  Asynchronous and real-time | | | | | ●●◯ MODERATE  IG (n=60), CG (n=29)  Asynchronous and real-time | | | |
| **Satisfaction** | **Fantinelli et al. 2019** IG highly satisfied (P=0.71, P<0.001) | | | **Lemelin et al. 2020**  Educational satisfaction:↑ in IG, P=0.028 | | | | | **Given et al. 2015**  IG=91%, CG=85%, SNA | | | | **Caballero-Ruiz et al. 2017**  IG highly satisfied, SNA | |
|  | ●●◯ MODERATE  n=831  SR | | | ●◯◯ LOW  IG (n=80), CG (n=81)  Asynchronous | | | | | ●●◯ MODERATE  IG (n=21), CG (n=26)  Asynchronous and real-time | | | | ●●◯ MODERATE  IG (n=60), CG (n=29)  Asynchronous and real-time | |
| **Diabates self-efficacy** | **Rasekaba et al. 2018**  Higher in IG in subscale 1: P=0.039 and subscale 2: P=0.036 | | | | | | **Fantinelli et al. 2019**  Higher in IG: overall P=0.053 [referring to same study as Rasekaba et al.) | | | | | | | |
|  | ●●◯ MODERATE  IG (n=32), CG (n=25)  Asynchronous | | | | | | ●●◯ MODERATE  IG (n=32), CG (n=25)  SR | | | | | | | |

^1^ Study quality in high, moderate or low, ^2^ Asynchronous: “Asynchronous communication” in intervention (internet/web-based), ^3^ Asynchronous and real-time: “Asynchronous and real-time communication” in intervention (internet/web-based and telephone calls)

CG = control group, HR = hazard ratio, IG = intervention group, IQR = interquartile range, MA = meta-analysis, OR = odds ratio, RR = relative risk, SD = standard deviation, SNA = significance not availabe

Table B-2: Effects on maternal complications in pregnancy and childbirth

| **Outcome** | **Study, effects, study quality, sample size, study type/intervention type** | | | | | | | | | | | |
| --- | --- | --- | --- | --- | --- | --- | --- | --- | --- | --- | --- | --- |
| **Pregnancy induced hypertension (RR or %)** | **Raman et al. 2017**  RR 1.49 (95% CI: 0.69, 3.20) | | **Perez-Ferre et al. 2010b**  IG=4.1%, CG=0%, P=0.501 | | | **Lemelin et al. 2020**  IG=1.3%, CG=2.5%, P=0.230 | | | | **Given et al. 2015**  IG=0.0%, IG=3.9%, SNA | | |
|  | **●●●** HIGH ^1^  n=275  SR/MA | | **●●●** HIGH  IG (n=49), CG (n=48)  Asynchronous ² | | | ●◯◯ LOW  IG (n=80), CG (n=81)  Asynchronous | | | | ●●◯ MODERATE  IG (n=21), CG (n=26)  Asynchronous and real-time ³ | | |
| **Pre-eclampsia (%)** | **Raman et al. 2017**  RR 1.49 (95% CI: 0.69, 3.20) | | **Lemelin et al. 2020**  IG=0%, CG=0% | | | **Homko et al. 2012**  IG=8%, CG=5%, P=0.7 | | | | **Given et al. 2015**  IG=0.0%, CG=3.9%, SNA | | |
|  | **●●●** HIGH  n=275  SR/MA | | ●◯◯ LOW  IG (n=80), CG (n=81)  Asynchronous | | | ●●◯ MODERATE  IG (n=36), CG (n=38)  Asynchronous and real-time | | | | ●●◯ MODERATE  IG (n=21), CG (n=26)  Asynchronous and real-time | | |
| **Caesarean section rate (%)** | **Rasekaba et al. 2015**  OR 0.48  (95% CI: 0.10, 2.35) | **Raman et al. 2017**  RR 1.05  (95% CI: 0.72, 1.53) | | **Rasekaba et al. 2018**  IG=46%  (95% CI: 33, 59)  CG= 32%  (95% CI: 17, 51) | **Perez-Ferre et al. 2010b**  IG=34.7% CG=25%  P=0.427 | | | **Lemelin et al. 2020**  IG=18.8%  CG=32.1%  P=0.070 | **Given et al. 2015**  IG=47.6%  CG=38.5%  SNA | | | **Homko et al. 2012**  IG=36%  CG=50%  P=0.3 |
|  | ●●◯ MODERATE  IG (n=117),  CG (n=111)  SR/MA | **●●●** HIGH  n=478  SR/MA | | ●●◯ MODERATE  IG (n=61), CG (n=34) Asynchronous | **●●●** HIGH  IG (n=49), CG (n=48)  Asynchronous | | | ●◯◯ LOW  IG (n=80), CG (n=81)  Asynchronous | ●●◯ MODERATE  IG (n=21), CG (n=26)  Asynchronous and  real-time | | | ●●◯ MODERATE  IG (n=36), CG (n=38)  Asynchronous and  real-time |
| **Preterm delivery  (<37 weeks) (%)** | **Perez-Ferre et al. 2010b**  IG=2.1%, CG=2.0%, P=0.500 | | **Lemelin et al. 2020** IG=3.8%, CG=0.0%, P=0.079 | | | | **Given et al. 2015** IG=0%, CG=8%, SNA | | | | **Homko et al. 2012** IG=5.6%, CG=13.2%, P=0.4 | |
|  | **●●●** HIGH  IG (n=49), CG (n=48)  Asynchronous | | ●◯◯ LOW  IG (n=80), CG (n=81)  Asynchronous | | | | ●●◯ MODERATE  IG (n=21), CG (n=26)  Asynchronous and real-time | | | | ●●◯ MODERATE  IG (n=36), CG (n=38)  Asynchronous and real-time | |
| **Induction of labour (RR)** | **Raman et al. 2017** **●●●** HIGH n=74 SR/MA | | | | | | | | | | | |
|  | RR 1.06 (95% CI: 0.63, 1.77) | | | | | | | | | | | |
| **Umbilical cord pathology (%)** | **Perez-Ferre et al. 2010b ●●●** HIGH IG (n=49), CG (n=48) Asynchronous | | | | | | | | | | | |
|  | IG=4.2%, CG=2.0%, P=0.500 | | | | | | | | | | | |
| **Abruptio placentae (%)** | **Perez-Ferre et al. 2010b ●●●** HIGH IG (n=49), CG (n=48) Asynchronous | | | | | | | | | | | |
|  | IG=2.1%, CG=0%, P=0.500 | | | | | | | | | | | |
| **Chorioamnionitis (%)** | **Homko et al. 2012** ●●◯ MODERATE IG (n=36), CG (n=38) Asynchronous and real-time | | | | | | | | | | | |
|  | IG=2.8%, CG=2.6%, P=1.0 | | | | | | | | | | | |

^1^ Study quality in high, moderate or low, ^2^ Asynchronous: “Asynchronous communication” in intervention (internet/web-based), ^3^ Asynchronous and real-time: “Asynchronous and real-time communication” in intervention (internet/web-based and telephone calls)
CG = control group, HR = hazard ratio, IG = intervention group, IQR = interquartile range, MA = meta-analysis, OR = odds ratio, RR = relative risk, SD = standard deviation, SNA = significance not availabe

Table B-3: Effects on fetal and neonatal short-term outcomes

| **Outcome** | **Study, effects, study quality, sample size, study type/intervention type** | | | | | | | | | |
| --- | --- | --- | --- | --- | --- | --- | --- | --- | --- | --- |
| **Biparietal diameter (cm)** | **Rasekaba et al. 2018** ●●◯ MODERATE ^1^ IG (n=61), CG (n=34) Asynchronous ² | | | | | | | | | |
|  | IG=8.4±0.6, CG=8.2±0.7, P=0.20 | | | | | | | | | |
| **Loss of fetal wellbeing (%)** | **Perez-Ferre et al. 2010b ●●●** HIGH IG (n=49), CG (n=48) Asynchronous | | | | | | | | | |
|  | IG=6.1, CG=8.3, P=0.500 | | | | | | | | | |
| **Intrauterine death (%)** | **Given et a. 2015** ●●◯ MODERATE IG (n=21), CG (n=26) Asynchronous and real-time ³ | | | | | | | | | |
|  | IG=0.0, CG=3.9, SNA | | | | | | | | | |
| **Large for gestational age (LGA) (RR or %)** | **Raman et al. 2017**  RR 1.41 (95% CI: 0.76, 2.64) | | | **Perez-Ferre et al. 2010b**  IG=6.1%, CC=8.3%, P=0.500 | | | | **Homko et al. 2012**  IG=25%, CG=18.4%, P=0.7 | | |
|  | **●●●** HIGH  n=228  SR/MA | | | **●●●** HIGH  IG (n=49), CG (n=48)  Asynchronous | | | | ●●◯ MODERATE  IG (n=36), CG (n=38)  Asynchronous and real-time | | |
| **Macrosomia  (≥4000g) (%)** | **Rasekaba et al. 2015**  Similar in groups (authors interpretation)  P>0.05 | | **Rasekaba et al. 2018**  IG=4.9% (95% CI: 1.0, 17.7)  CG=2.9% (95% CI: 0.1, 15.3), P=1.00 | | | | **Lemelin et al. 2020**  IG=1.3%, CG=2.5%, P=1.000 | | **Given et al. 2015**  IG=28.6%, CG=8%, SNA | |
|  | ●●◯ MODERATE  IG (n=117), CG (n=111)  SR/MA | | ●●◯ MODERATE  IG (n=61), CG (n=34) Asynchronous | | | | ●◯◯ LOW  IG (n=80), CG (n=81)  Asynchronous | | ●●◯ MODERATE  IG (n=21), CG (n=26)  Asynchronous and real-time | |
| **Birth weight (g)** | **Rasekaba et al. 2015**  Similar in groups (authors interpretation)  P>0.05 | **Rasekaba et al. 2018**  IG=3311g±455 CG=3275g±384  P=0.69 | | | **Perez-Ferre et al. 2010b**  IG=3308.2g±488.8  CG=3370.6g±479.1  P=0.385 | **Lemelin et al. 2020**  IG=3263g±336  CG=3292g±391  P=0.608 | | **Given et al. 2015**  IG=3557g±599  CG=3272g±443  SNA | | **Homko et al. 2012**  IG=3372g±469  CG=3249g±611 P=0.3 |
|  | ●●◯ MODERATE  IG (n=117), CG (n=111)  SR/MA | ●●◯ MODERATE  IG (n=61), CG (n=34) Asynchronous | | | **●●●** HIGH  IG (n=49), CG (n=48)  Asynchronous | ●◯◯ LOW  IG (n=80), CG (n=81)  Asynchronous | | ●●◯ MODERATE  IG (n=21), CG (n=26)  Asynchronous and  real-time | | ●●◯ MODERATE  IG (n=36), CG (n=38)  Asynchronous and  real-time |
| **Respiratory distress syndrome (%)** | **Lemelin et al. 2020**  IG=1.3%, CG=1.2%, P=1.000 | | | | **Given et al. 2015**  IG=4.0%, CG=15.0%, SNA | | | **Homko et al. 2012**  IG=5.6% , CG=13.2%, P=0.4 | | |
|  | ●◯◯ LOW  IG (n=80), CG (n=81)  Asynchronous | | | | ●●◯ MODERATE  IG (n=21), CG (n=26)  Asynchronous and real-time | | | ●●◯ MODERATE  IG (n=36), CG (n=38)  Asynchronous and real-time | | |
| **Shoulder dystocia (%)** | **Perez-Ferre et al. 2010b**  IG=0.0%, CG=2.1%, P=0.500 | | | | **Lemelin et al. 2020**  IG=2.5, CG=0.0, P=0.245 | | | **Given et al. 2015**  IG=0.0, CG=0.0, SNA | | |
|  | **●●●** HIGH  IG (n=49), CG (n=48)  Asynchronous | | | | ●◯◯ LOW  IG (n=80), CG (n=81)  Asynchronous | | | ●●◯ MODERATE  IG (n=21), CG (n=26)  Asynchronous and real-time | | |
| **Admitted to neonatal intensive care unit (%)** | **Given et al. 2015**  IG=36, CG=45, SNA | | | | | **Homko et al. 2012**  IG=11%, CG=18.4%, P=0.6 | | | | |
|  | ●●◯ MODERATE  IG (n=21), CG (n=26)  Asynchronous and real-time | | | | | ●●◯ MODERATE  IG (n=36), CG (n=38)  Asynchronous and real-time | | | | |

^1^ Study quality in high, moderate or low, ^2^ Asynchronous: “Asynchronous communication” in intervention (internet/web-based), ^3^ Asynchronous and real-time: “Asynchronous and real-time communication” in intervention (internet/web-based and telephone calls)

CG = control group, HR = hazard ratio, IG = intervention group, IQR = interquartile range, MA = meta-analysis, OR = odds ratio, RR = relative risk, SD = standard deviation, SNA = significance
